# Side locked headaches

**DOI:** 10.1186/1129-2377-14-S1-P64

**Published:** 2013-02-21

**Authors:** A Momoh-Ojewuyi, A Ayubi, R Abusamra, S Al-Ani, A Whitehouse, R Alkilani, MAS Ahmed

**Affiliations:** 1 Barking Havering & Redbridge NHS Trust, UK

## Methods

Data were prospectively collected from 975 eligible patients (554 females; 583 Caucasians; age range = 4.5-18.1 years) with headaches. Patients were included only if they were > 4 years old and had suffered headache course for > 6 months and or 5 separate headache attacks. We have adopted previous descriptions of terms for anatomical sites for location [1]. Side locked unilateral headache (SLUH) is defined as a headache that is for all time fixed unilaterally and never changed side. Headache diagnosis was made on the basis of ICHD – II, 2004 [2]. Headache diagnosis included migraine (n=585); tension type headaches (n= 234); other headache types (n=91) and remained unclassified in 65 (7%) patients.

## Results

119/975 (12%) of patients experienced recurrent SLUH during a mean headache course of 2.3 years. It was more for unilateral SLUH to localise to the right than the left (60% vs 40%). Topographically, temporal headache was the most frequent, followed by frontal and then parietal. Headaches were SLUH in 11.5% of patients with migraine; 8% with TTH and 23% patients with headache that not yet specified. Brain imaging was normal or showed no significant abnormalities in all scanned patients.

## Discussion

Sinister aetiologies of SLUH were excluded among our patients. Primary headache was the most common headache category among patients with SLUH. Although, migraine constituted 60% of our study series, frequency of SLUH among migraineurs and those with non-migraine headaches did not reach statistical significance (11.5% vs 13%).

## Conclusion

Before one could reach a conclusion of sinister aetiologies when faced with a patient with SLUH, primary headaches such as migraine and TTH should be considered.

## References

[B1] Chakravarty A J Headache Pain 2008 14 375 379 10.1007/s10194-008-0075-1 18854921 PMC3452085

[B2] International Headache Society ICHD-II Cephalalgia 2004 14 Suppl 1

